# 1,2,3,4-Tetra­methyl­cyclo­pent-2-ene-1,4-diol

**DOI:** 10.1107/S160053680802775X

**Published:** 2008-09-06

**Authors:** Steffen Blaurock, Axel Fischer, Jochen Gottfriedsen, Marlies Spoida, Frank T. Edelmann

**Affiliations:** a Chemisches Institut, Otto-von-Guericke-Universität Magdeburg, Universitätsplatz 2, D-39106 Magdeburg, Germany

## Abstract

The title compound, C_9_H_16_O_2_, crystallizes with two mol­ecules in the asymmetric unit. The structure displays inter­molecular O—H⋯O hydrogen bonding.

## Related literature

For related literature, see: Etter (1991 ▶); Brock & Duncan (1994 ▶); Fendrick *et al.* (1988 ▶).
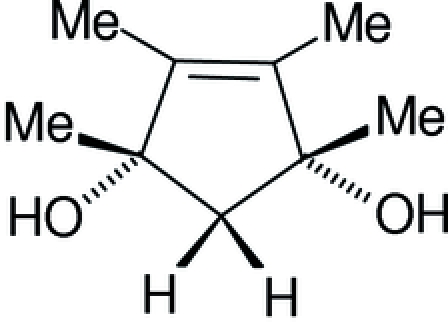


## Experimental

### 

#### Crystal data

C_9_H_16_O_2_
*M* *_r_* = 156.22
Monoclinic, 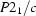

*a* = 13.006 (3) Å
*b* = 10.5279 (16) Å
*c* = 13.892 (2) Å
β = 107.257 (10)°
*V* = 1816.6 (6) Å^3^
*Z* = 8
Mo *K*α radiation
μ = 0.08 mm^−1^
*T* = 133 (2) K
0.45 × 0.28 × 0.07 mm

#### Data collection

Bruker SMART CCD diffractometer
Absorption correction: none
20936 measured reflections
5532 independent reflections
3876 reflections with *I* > 2σ(*I*)
*R*_int_ = 0.048

#### Refinement

*R*[*F* ^2^ > 2σ(*F* ^2^)] = 0.048
*wR*(*F* ^2^) = 0.142
*S* = 1.03
5532 reflections
223 parameters
H atoms treated by a mixture of independent and constrained refinement
Δρ_max_ = 0.41 e Å^−3^
Δρ_min_ = −0.23 e Å^−3^

### 

Data collection: *SMART* (Bruker, 1997 ▶); cell refinement: *SAINT* (Bruker, 1997 ▶); data reduction: *SAINT*; program(s) used to solve structure: *SHELXS97* (Sheldrick, 2008 ▶); program(s) used to refine structure: *SHELXL97* (Sheldrick, 2008 ▶); molecular graphics: *XP5* in *SHELXTL* (Sheldrick, 2008 ▶); software used to prepare material for publication: *SHELXL97*.

## Supplementary Material

Crystal structure: contains datablocks I, global. DOI: 10.1107/S160053680802775X/bt2745sup1.cif

Structure factors: contains datablocks I. DOI: 10.1107/S160053680802775X/bt2745Isup2.hkl

Additional supplementary materials:  crystallographic information; 3D view; checkCIF report

## Figures and Tables

**Table 1:** Hydrogen-bond geometry (Å, °)

*D*—H⋯*A*	*D*—H	H⋯*A*	*D*⋯*A*	*D*—H⋯*A*
O1—H01⋯O2^i^	0.95 (2)	1.80 (2)	2.7438 (13)	174.7 (17)
O1′—H01′⋯O1	0.921 (18)	1.824 (18)	2.7345 (13)	169.3 (16)
O2—H02⋯O2′^ii^	0.841 (19)	1.89 (2)	2.7263 (13)	173.0 (18)
O2′—H02′⋯O1′^ii^	0.86 (2)	1.89 (2)	2.7333 (13)	166.1 (18)

Symmetry codes: (i) 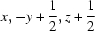; (ii) 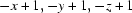.

**Fig. 1.**
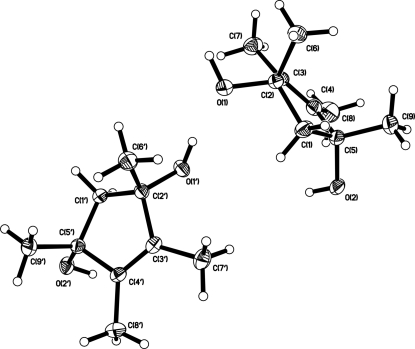
The molecule of the title compound in the crystal. Displacement ellipsoids represent 50% probability levels. H-Atom radii are arbitrary.

**Fig. 2.**
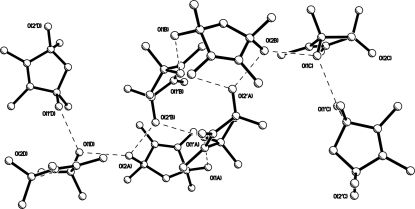
The hydrogen-bond network.

## supplementary crystallographic information

### Comment

In the solid state, alcohols generally form hydrogen-bonded networks resulting in a variety of ring, chain, or helix structures (Brock & Duncan, 1994). The hitherto unknown title compound, 1,2,3,4-tetramethylcyclopen-2-ene-1,4-diol, was obtained in minor quantities (less than 5% isolated yield) in the form of colorless crystals during a preparation of 1,2,3,4-tetramethylcyclopentadiene according to the literature (Fendrick *et al.*, 1988). The structure of the title compound is shown in Figure 1. Dimensions are available in the archived CIF. Especially notable is the hydrogen-bond network in the crystal structure. As depicted in Figure 2, four molecules of 1,2,3,4-tetramethylcyclopen-2-ene-1,4-diol are connected *via* hydrogen-bonds to give cyclic tetramers. Further hydrogen-bonding between adjacent tetrameric units results in an extended hydrogen-bond network.

### Experimental

Crystals of the title compound were obtained as a minor by-product during the synthesis of 1,2,3,4-tetramethylcyclopentadiene according to the literature preparatio (Fendrick *et al.*, 1988).

### Refinement

H atoms bonded to C were refined with fixed individual displacement parameters [U(H) = 1.2 U_eq_(C) or U(H) = 1.5 U_eq_(C_methyl_)] using a riding model with C-H(methylen) = 0.99 Å or C-H(methyl) = 0.98Å, respectively. The H atoms bonded to   O were refined isotropically.

### Crystal data

**Table:**

C_9_H_16_O_2_	*F*(000) = 688
*M**_r_* = 156.22	*D*_x_ = 1.142 Mg m^−^^3^
Monoclinic, *P*2_1_/*c*	Mo *K*α radiation, λ = 0.71073 Å
Hall symbol: -P2ybc	Cell parameters from 5784 reflections
*a* = 13.006 (3) Å	θ = 2.5–30.0°
*b* = 10.5279 (16) Å	µ = 0.08 mm^−^^1^
*c* = 13.892 (2) Å	*T* = 133 K
β = 107.257 (10)°	Plate, colourless
*V* = 1816.6 (6) Å^3^	0.45 × 0.28 × 0.07 mm
*Z* = 8	

### Data collection

**Table:**

Bruker SMART CCD diffractometer	3876 reflections with *I* > 2σ(*I*)
Radiation source: fine-focus sealed tube	*R*_int_ = 0.048
graphite	θ_max_ = 30.5°, θ_min_ = 1.6°
Detector resolution: 8.192 pixels mm^-1^	*h* = −18→18
ω–scans	*k* = −14→15
20936 measured reflections	*l* = −19→19
5532 independent reflections	

### Refinement

**Table:**

Refinement on *F*^2^	Primary atom site location: structure-invariant direct methods
Least-squares matrix: full	Secondary atom site location: difference Fourier map
*R*[*F*^2^ > 2σ(*F*^2^)] = 0.048	Hydrogen site location: inferred from neighbouring sites
*wR*(*F*^2^) = 0.142	H atoms treated by a mixture of independent and constrained refinement
*S* = 1.03	*w* = 1/[σ^2^(*F*_o_^2^) + (0.0812*P*)^2^] where *P* = (*F*_o_^2^ + 2*F*_c_^2^)/3
5532 reflections	(Δ/σ)_max_ = 0.002
223 parameters	Δρ_max_ = 0.41 e Å^−^^3^
0 restraints	Δρ_min_ = −0.23 e Å^−^^3^

### Special details

**Table:**

Geometry. All e.s.d.'s (except the e.s.d. in the dihedral angle between two l.s. planes) are estimated using the full covariance matrix. The cell e.s.d.'s are taken into account individually in the estimation of e.s.d.'s in distances, angles and torsion angles; correlations between e.s.d.'s in cell parameters are only used when they are defined by crystal symmetry. An approximate (isotropic) treatment of cell e.s.d.'s is used for estimating e.s.d.'s involving l.s. planes.
Refinement. Refinement of *F*^2^ against ALL reflections. The weighted *R*-factor *wR* and goodness of fit *S* are based on *F*^2^, conventional *R*-factors *R* are based on *F*, with *F* set to zero for negative *F*^2^. The threshold expression of *F*^2^ > σ(*F*^2^) is used only for calculating *R*-factors(gt) *etc*. and is not relevant to the choice of reflections for refinement. *R*-factors based on *F*^2^ are statistically about twice as large as those based on *F*, and *R*- factors based on ALL data will be even larger.

### Fractional atomic coordinates and isotropic or equivalent isotropic displacement parameters (Å^2^)

**Table:**

	*x*	*y*	*z*	*U*_iso_*/*U*_eq_	
O1	0.16035 (7)	0.34338 (9)	0.59028 (7)	0.0255 (2)	
O2	0.16763 (8)	0.21564 (10)	0.28457 (7)	0.0330 (3)	
H02	0.2279 (16)	0.2415 (16)	0.3207 (14)	0.048 (5)*	
H01	0.1585 (14)	0.3212 (17)	0.6560 (15)	0.055 (5)*	
C1	0.08579 (10)	0.28972 (13)	0.41367 (9)	0.0260 (3)	
H1A	0.0100	0.3172	0.3852	0.031*	
H1B	0.1335	0.3623	0.4115	0.031*	
C2	0.10655 (9)	0.24265 (12)	0.52290 (8)	0.0210 (2)	
C3	0.17851 (9)	0.12814 (12)	0.52756 (9)	0.0217 (2)	
C4	0.17849 (10)	0.09054 (12)	0.43564 (10)	0.0242 (3)	
C5	0.11007 (10)	0.17648 (13)	0.35382 (9)	0.0260 (3)	
C6	0.00335 (10)	0.20778 (14)	0.54767 (10)	0.0290 (3)	
H6C	0.0216	0.1724	0.6159	0.035*	
H6B	−0.0368	0.1446	0.4992	0.035*	
H6A	−0.0410	0.2840	0.5438	0.035*	
C7	0.23648 (11)	0.06809 (14)	0.62666 (10)	0.0312 (3)	
H7C	0.2703	−0.0112	0.6148	0.037*	
H7B	0.1851	0.0499	0.6641	0.037*	
H7A	0.2920	0.1263	0.6659	0.037*	
C8	0.23380 (13)	−0.02192 (15)	0.40823 (12)	0.0397 (4)	
H8C	0.2779	−0.0635	0.4698	0.048*	
H8B	0.2800	0.0058	0.3678	0.048*	
H8A	0.1799	−0.0819	0.3691	0.048*	
C9	0.00891 (12)	0.11076 (17)	0.28879 (11)	0.0403 (4)	
H9C	−0.0333	0.1707	0.2385	0.048*	
H9B	−0.0343	0.0814	0.3315	0.048*	
H9A	0.0293	0.0379	0.2545	0.048*	
O1'	0.34071 (7)	0.44079 (8)	0.55499 (7)	0.0229 (2)	
H01'	0.2803 (14)	0.4165 (17)	0.5720 (13)	0.042 (5)*	
O2'	0.63799 (7)	0.68485 (9)	0.61022 (7)	0.0229 (2)	
H02'	0.6360 (15)	0.6373 (17)	0.5589 (15)	0.053 (5)*	
C1'	0.48169 (9)	0.58556 (11)	0.64713 (8)	0.0188 (2)	
H1'1	0.5235	0.5089	0.6413	0.023*	
H1'2	0.4843	0.5963	0.7186	0.023*	
C2'	0.36462 (9)	0.57287 (11)	0.58020 (9)	0.0183 (2)	
C3'	0.36529 (9)	0.64743 (11)	0.48658 (9)	0.0195 (2)	
C4'	0.45363 (9)	0.71901 (11)	0.50268 (8)	0.0190 (2)	
C5'	0.52810 (9)	0.70382 (11)	0.60928 (8)	0.0182 (2)	
C6'	0.28221 (10)	0.62512 (13)	0.62934 (10)	0.0258 (3)	
H6'C	0.2096	0.6166	0.5823	0.031*	
H6'B	0.2974	0.7149	0.6461	0.031*	
H6'A	0.2869	0.5772	0.6910	0.031*	
C7'	0.27255 (10)	0.63672 (14)	0.39236 (10)	0.0279 (3)	
H7'C	0.2824	0.6968	0.3420	0.033*	
H7'B	0.2054	0.6563	0.4076	0.033*	
H7'A	0.2691	0.5501	0.3658	0.033*	
C8'	0.48398 (11)	0.80742 (12)	0.43083 (10)	0.0269 (3)	
H8'C	0.4254	0.8108	0.3674	0.032*	
H8'B	0.5497	0.7766	0.4178	0.032*	
H8'A	0.4966	0.8926	0.4604	0.032*	
C9'	0.52987 (11)	0.82140 (12)	0.67418 (10)	0.0251 (3)	
H9'C	0.5805	0.8079	0.7414	0.030*	
H9'B	0.4576	0.8364	0.6801	0.030*	
H9'A	0.5526	0.8953	0.6427	0.030*	

### Atomic displacement parameters (Å^2^)

**Table:**

	*U*^11^	*U*^22^	*U*^33^	*U*^12^	*U*^13^	*U*^23^
O1	0.0289 (5)	0.0282 (5)	0.0219 (4)	−0.0072 (4)	0.0112 (4)	−0.0031 (4)
O2	0.0267 (5)	0.0570 (7)	0.0158 (4)	−0.0163 (5)	0.0073 (4)	−0.0031 (4)
C1	0.0231 (6)	0.0341 (7)	0.0202 (6)	0.0010 (5)	0.0056 (5)	0.0049 (5)
C2	0.0199 (6)	0.0263 (6)	0.0171 (5)	−0.0033 (5)	0.0058 (4)	0.0005 (4)
C3	0.0188 (6)	0.0260 (6)	0.0205 (6)	−0.0037 (5)	0.0061 (5)	0.0017 (5)
C4	0.0226 (6)	0.0274 (6)	0.0243 (6)	−0.0055 (5)	0.0095 (5)	−0.0036 (5)
C5	0.0208 (6)	0.0402 (7)	0.0173 (6)	−0.0091 (5)	0.0061 (5)	−0.0010 (5)
C6	0.0216 (6)	0.0397 (8)	0.0278 (7)	−0.0046 (5)	0.0106 (5)	−0.0011 (5)
C7	0.0316 (7)	0.0343 (7)	0.0265 (7)	0.0029 (6)	0.0067 (6)	0.0063 (6)
C8	0.0442 (9)	0.0363 (8)	0.0449 (9)	0.0000 (7)	0.0229 (7)	−0.0084 (7)
C9	0.0297 (7)	0.0629 (11)	0.0264 (7)	−0.0213 (7)	0.0055 (6)	−0.0049 (7)
O1'	0.0221 (4)	0.0214 (4)	0.0278 (5)	−0.0045 (3)	0.0114 (4)	−0.0053 (3)
O2'	0.0167 (4)	0.0300 (5)	0.0219 (4)	−0.0034 (3)	0.0057 (3)	−0.0059 (4)
C1'	0.0199 (5)	0.0204 (5)	0.0160 (5)	−0.0008 (4)	0.0053 (4)	0.0006 (4)
C2'	0.0185 (5)	0.0182 (5)	0.0193 (5)	−0.0014 (4)	0.0074 (4)	−0.0025 (4)
C3'	0.0204 (5)	0.0211 (6)	0.0166 (5)	0.0036 (4)	0.0049 (4)	−0.0002 (4)
C4'	0.0227 (6)	0.0192 (5)	0.0161 (5)	0.0025 (4)	0.0071 (4)	0.0009 (4)
C5'	0.0171 (5)	0.0206 (6)	0.0175 (5)	−0.0010 (4)	0.0062 (4)	−0.0009 (4)
C6'	0.0249 (6)	0.0274 (6)	0.0288 (6)	−0.0009 (5)	0.0138 (5)	−0.0038 (5)
C7'	0.0250 (6)	0.0323 (7)	0.0215 (6)	0.0025 (5)	−0.0006 (5)	0.0001 (5)
C8'	0.0320 (7)	0.0270 (6)	0.0241 (6)	0.0014 (5)	0.0119 (5)	0.0067 (5)
C9'	0.0301 (6)	0.0237 (6)	0.0230 (6)	−0.0028 (5)	0.0101 (5)	−0.0054 (5)

### Geometric parameters (Å, °)

**Table:**

O1—C2	1.4498 (14)	O1'—C2'	1.4451 (14)
O1—H01	0.95 (2)	O1'—H01'	0.921 (18)
O2—C5	1.4434 (15)	O2'—C5'	1.4394 (14)
O2—H02	0.841 (19)	O2'—H02'	0.86 (2)
C1—C5	1.5388 (19)	C1'—C2'	1.5365 (16)
C1—C2	1.5421 (17)	C1'—C5'	1.5425 (16)
C1—H1A	0.9900	C1'—H1'1	0.9900
C1—H1B	0.9900	C1'—H1'2	0.9900
C2—C3	1.5157 (18)	C2'—C3'	1.5214 (16)
C2—C6	1.5259 (17)	C2'—C6'	1.5334 (17)
C3—C4	1.3369 (17)	C3'—C4'	1.3362 (17)
C3—C7	1.4992 (17)	C3'—C7'	1.4981 (17)
C4—C8	1.492 (2)	C4'—C8'	1.5007 (17)
C4—C5	1.5174 (19)	C4'—C5'	1.5184 (16)
C5—C9	1.5233 (18)	C5'—C9'	1.5275 (16)
C6—H6C	0.9800	C6'—H6'C	0.9800
C6—H6B	0.9800	C6'—H6'B	0.9800
C6—H6A	0.9800	C6'—H6'A	0.9800
C7—H7C	0.9800	C7'—H7'C	0.9800
C7—H7B	0.9800	C7'—H7'B	0.9800
C7—H7A	0.9800	C7'—H7'A	0.9800
C8—H8C	0.9800	C8'—H8'C	0.9800
C8—H8B	0.9800	C8'—H8'B	0.9800
C8—H8A	0.9800	C8'—H8'A	0.9800
C9—H9C	0.9800	C9'—H9'C	0.9800
C9—H9B	0.9800	C9'—H9'B	0.9800
C9—H9A	0.9800	C9'—H9'A	0.9800
			
C2—O1—H01	107.2 (11)	C2'—O1'—H01'	110.1 (11)
C5—O2—H02	105.7 (13)	C5'—O2'—H02'	106.6 (13)
C5—C1—C2	106.18 (10)	C2'—C1'—C5'	106.36 (9)
C5—C1—H1A	110.5	C2'—C1'—H1'1	110.5
C2—C1—H1A	110.5	C5'—C1'—H1'1	110.5
C5—C1—H1B	110.5	C2'—C1'—H1'2	110.5
C2—C1—H1B	110.5	C5'—C1'—H1'2	110.5
H1A—C1—H1B	108.7	H1'1—C1'—H1'2	108.6
O1—C2—C3	112.37 (10)	O1'—C2'—C3'	110.16 (9)
O1—C2—C6	108.58 (10)	O1'—C2'—C6'	108.94 (10)
C3—C2—C6	111.86 (10)	C3'—C2'—C6'	112.18 (10)
O1—C2—C1	108.08 (10)	O1'—C2'—C1'	109.49 (9)
C3—C2—C1	102.90 (10)	C3'—C2'—C1'	102.50 (9)
C6—C2—C1	112.97 (10)	C6'—C2'—C1'	113.42 (10)
C4—C3—C7	127.58 (12)	C4'—C3'—C7'	128.18 (11)
C4—C3—C2	111.69 (11)	C4'—C3'—C2'	111.76 (10)
C7—C3—C2	120.70 (11)	C7'—C3'—C2'	120.05 (11)
C3—C4—C8	127.96 (13)	C3'—C4'—C8'	128.40 (11)
C3—C4—C5	111.89 (11)	C3'—C4'—C5'	111.83 (10)
C8—C4—C5	120.13 (12)	C8'—C4'—C5'	119.76 (10)
O2—C5—C4	111.39 (11)	O2'—C5'—C4'	111.58 (9)
O2—C5—C9	105.18 (10)	O2'—C5'—C9'	105.43 (9)
C4—C5—C9	112.63 (12)	C4'—C5'—C9'	112.58 (10)
O2—C5—C1	111.66 (11)	O2'—C5'—C1'	111.89 (9)
C4—C5—C1	103.06 (10)	C4'—C5'—C1'	102.51 (9)
C9—C5—C1	113.13 (12)	C9'—C5'—C1'	113.06 (10)
C2—C6—H6C	109.5	C2'—C6'—H6'C	109.5
C2—C6—H6B	109.5	C2'—C6'—H6'B	109.5
H6C—C6—H6B	109.5	H6'C—C6'—H6'B	109.5
C2—C6—H6A	109.5	C2'—C6'—H6'A	109.5
H6C—C6—H6A	109.5	H6'C—C6'—H6'A	109.5
H6B—C6—H6A	109.5	H6'B—C6'—H6'A	109.5
C3—C7—H7C	109.5	C3'—C7'—H7'C	109.5
C3—C7—H7B	109.5	C3'—C7'—H7'B	109.5
H7C—C7—H7B	109.5	H7'C—C7'—H7'B	109.5
C3—C7—H7A	109.5	C3'—C7'—H7'A	109.5
H7C—C7—H7A	109.5	H7'C—C7'—H7'A	109.5
H7B—C7—H7A	109.5	H7'B—C7'—H7'A	109.5
C4—C8—H8C	109.5	C4'—C8'—H8'C	109.5
C4—C8—H8B	109.5	C4'—C8'—H8'B	109.5
H8C—C8—H8B	109.5	H8'C—C8'—H8'B	109.5
C4—C8—H8A	109.5	C4'—C8'—H8'A	109.5
H8C—C8—H8A	109.5	H8'C—C8'—H8'A	109.5
H8B—C8—H8A	109.5	H8'B—C8'—H8'A	109.5
C5—C9—H9C	109.5	C5'—C9'—H9'C	109.5
C5—C9—H9B	109.5	C5'—C9'—H9'B	109.5
H9C—C9—H9B	109.5	H9'C—C9'—H9'B	109.5
C5—C9—H9A	109.5	C5'—C9'—H9'A	109.5
H9C—C9—H9A	109.5	H9'C—C9'—H9'A	109.5
H9B—C9—H9A	109.5	H9'B—C9'—H9'A	109.5
			
C5—C1—C2—O1	139.28 (10)	C5'—C1'—C2'—O1'	138.54 (9)
C5—C1—C2—C3	20.24 (12)	C5'—C1'—C2'—C3'	21.61 (11)
C5—C1—C2—C6	−100.55 (12)	C5'—C1'—C2'—C6'	−99.55 (11)
O1—C2—C3—C4	−130.31 (11)	O1'—C2'—C3'—C4'	−130.65 (11)
C6—C2—C3—C4	107.25 (12)	C6'—C2'—C3'—C4'	107.81 (12)
C1—C2—C3—C4	−14.31 (13)	C1'—C2'—C3'—C4'	−14.19 (13)
O1—C2—C3—C7	51.62 (15)	O1'—C2'—C3'—C7'	50.55 (14)
C6—C2—C3—C7	−70.82 (14)	C6'—C2'—C3'—C7'	−70.99 (14)
C1—C2—C3—C7	167.63 (11)	C1'—C2'—C3'—C7'	167.00 (10)
C7—C3—C4—C8	1.6 (2)	C7'—C3'—C4'—C8'	−0.3 (2)
C2—C3—C4—C8	−176.35 (12)	C2'—C3'—C4'—C8'	−178.94 (11)
C7—C3—C4—C5	−179.83 (12)	C7'—C3'—C4'—C5'	179.35 (11)
C2—C3—C4—C5	2.27 (15)	C2'—C3'—C4'—C5'	0.67 (14)
C3—C4—C5—O2	130.67 (12)	C3'—C4'—C5'—O2'	133.00 (10)
C8—C4—C5—O2	−50.59 (16)	C8'—C4'—C5'—O2'	−47.36 (14)
C3—C4—C5—C9	−111.43 (13)	C3'—C4'—C5'—C9'	−108.69 (12)
C8—C4—C5—C9	67.31 (16)	C8'—C4'—C5'—C9'	70.96 (14)
C3—C4—C5—C1	10.82 (14)	C3'—C4'—C5'—C1'	13.10 (13)
C8—C4—C5—C1	−170.44 (12)	C8'—C4'—C5'—C1'	−167.25 (10)
C2—C1—C5—O2	−138.72 (10)	C2'—C1'—C5'—O2'	−140.96 (9)
C2—C1—C5—C4	−19.06 (12)	C2'—C1'—C5'—C4'	−21.28 (11)
C2—C1—C5—C9	102.85 (12)	C2'—C1'—C5'—C9'	100.18 (11)

### Hydrogen-bond geometry (Å, °)

**Table:**

*D*—H···*A*	*D*—H	H···*A*	*D*···*A*	*D*—H···*A*
O1—H01···O2^i^	0.95 (2)	1.80 (2)	2.7438 (13)	174.7 (17)
O1'—H01'···O1	0.921 (18)	1.824 (18)	2.7345 (13)	169.3 (16)
O2—H02···O2'^ii^	0.841 (19)	1.89 (2)	2.7263 (13)	173.0 (18)
O2'—H02'···O1'^ii^	0.86 (2)	1.89 (2)	2.7333 (13)	166.1 (18)

Symmetry codes: (i) *x*, −*y*+1/2, *z*+1/2; (ii) −*x*+1, −*y*+1, −*z*+1.
